# Autogenous Healing in Cementitious Materials with Superabsorbent Polymers Quantified by Means of NMR

**DOI:** 10.1038/s41598-020-57555-0

**Published:** 2020-01-20

**Authors:** D. Snoeck, L. Pel, N. De Belie

**Affiliations:** 1 Magnel Laboratory for Concrete Research, Department of Structural Engineering and Building Materials, Faculty of Engineering and Architecture, Ghent University, Tech Lane Ghent Science Park, Campus A, Technologiepark Zwijnaarde 60, B-9052 Gent, Belgium; 2 Transport in Permeable Media, Department of Applied Physics, Eindhoven University of Technology, Eindhoven, The Netherlands

**Keywords:** Civil engineering, Techniques and instrumentation, Techniques and instrumentation

## Abstract

A recent advance in construction technology is the use of self-healing cementitious materials containing synthetic microfibers and superabsorbent polymers. By stimulating autogenous healing by means of superabsorbent polymers, cracks are closed and this will cause an increase in durability and service life. However, this improved healing capacity has not been quantified yet in terms of increased further hydration and volume of healing products. This is needed to model the material and to stimulate the practical application in constructions. This paper provides quantitative data, obtained by an NMR study. Addition of 1 m% of selected superabsorbent polymer versus cement to a cementitious material, stimulated further hydration with nearly 40% in comparison with a traditional cementitious material, if 1 h water contact per day was allowed. At 90% relative humidity, no healing was observed in reference samples. While the further hydration around a crack in specimens with superabsorbent polymers was still 68% of that of a reference system with cyclic water contact, due to the uptake of moisture by the superabsorbent polymers. As such, NMR results quantify the positive influence of superabsorbent polymers in terms of stimulated autogenous healing and substantiate their benefits for application in the construction area.

## Introduction

Due to an increased interest in environmental issues, cementitious materials have to become more durable and sustainable. They need to show a reduced impact on the environment and subsequently an increased service life is needed. As concrete is susceptible to crack formation causing a decrease in durability and service life, the detrimental impact of cracks needs to be tackled. Manual repair is costly, labor-intensive and moreover not all parts of a structure are accessible due to the structure’s design and/or location. Recent advances in concrete research have led to self-healing concrete^1^. Through a dedicated design of the cementitious material, the autogenous healing capacity may be optimally used to serve as a solution to heal cracks and thereby increase the service life.

Through limiting the crack widths by means of synthetic microfibers, the formation of autogenous healing materials may bridge a crack by further hydration, pozzolanic activity and calcium carbonate crystallization^2,3^. For autogenous healing mechanisms, water needs to be present and this is not always the case. A possible solution for this problem is the use of superabsorbent polymers (SAPs)^4–6^. These materials are able to adsorb moisture and to absorb fluids from the surroundings, triggering the autogenous healing mechanisms as this water is released towards the building blocks for autogenous healing. Previous results showed a stimulated crack closure capacity and increase in mechanical properties upon reloading the healed material^7^. Even repeated reloading of cracked specimens showed a still present ability of superabsorbent polymers to promote autogenous healing^8^. As cracks are healed, they will cause less ingress of fluids and this may increase the durability in time and may lead to an increase in service life^9,10^. Even up to a testing age of 8 years, the specimens with superabsorbent polymers show an increased visual healing and a regain in mechanical properties compared to reference systems, both in wet/dry cycling as upon storage at high relative humidity^11^.

As superabsorbent polymers are able to trigger self-healing mechanisms due to adsorption and absorption, it is interesting to study the differences in terms of further hydration and to quantify the increase in healing ability compared to a reference system. It is, however, very difficult to quantify the healing capacity without destroying the material^12^. By using a non-destructive technique while subsequently healing the specimens, more information would be obtained. One way to study water transport and binding non-destructively in a hardening sample is the use of NMR. Some studies used NMR to study the hydration of cement^13–15^, the internal curing by superabsorbent polymers^16–18^, or drying and heating^19–23^. One study used a solid-state NMR to study the reactivity of sodium silicate based systems^24^. Using NMR the autogenous healing capacity in cement pastes was studied but not the effect of superabsorbent polymers^25^. The authors studied a cement paste prism with a hollow tube through which water could be added. It was found that water migrated from cracks into the bulk paste during autogenous healing. The amount of non-chemically bound water decreased indicating that some additional hydration occurred^25^.

**Figure 1:**
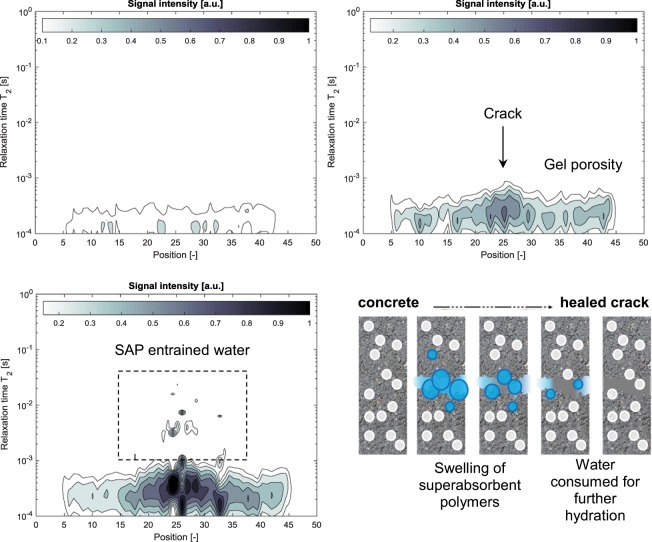
Typical recorded NMR signal intensity [a.u.] plot as a function of the vertical position and the T_2_ relaxation time for a dry reference sample (top left), a wet reference sample (top right), a wet sample containing superabsorbent polymers (bottom left) and the general principle of autogenous healing in cementitious materials by means of superabsorbent polymers (bottom right).

In this paper, NMR was used to study the promoted healing capacity by superabsorbent polymers. Typical NMR intensities as a function of the position (height) of the sample and the relaxation times T_2_ spectrum representing the pore water distribution as obtained by Fast Laplace Inversion can be found in Fig. 1. The dry reference sample contains gel and free capillary water in the range of 1·10^−4^ to 4·10^−4^ s. The signal is rather smeared due to the use of the cement containing iron oxides. When looking at an NMR scan of a wet reference sample, the intensities are higher, due to more free and available water. At 25 mm, the location of the crack in the middle of the specimen, a local increase is found. This corresponds to water found in the crack, as well as at the crack faces in filled accessible porosity. Sometimes larger relative signal intensities are found at locations other than the crack. These regions have some air pores near the sample surface, which are filled with water.

A typical wet specimen containing superabsorbent polymers has a higher total NMR signal intensity compared to a wet reference sample, mainly due to the storage of water in the macro pores containing superabsorbent polymers. The crack is clearly noticed at 26 mm and a possible macro pore or big capillary pore is seen at 33 mm. The same range of gel pores and capillary pores is found, with some signals for larger capillary pores near 1·10^−3^ s. Even slower relaxation times (1·10^−3^ s to 1·10^−2^ s) are found and are appointed to entrained water in superabsorbent polymers^16–18^. The NMR signals found for superabsorbent polymer water are not corresponding to the expected T_2_ relaxation times for such size of macro pores in the order of hundreds of micrometers. The values are variable and this points to a heterogeneous nature of the interior of the superabsorbent polymers. A possible explanation is related to an enhanced relaxation. This can be due to the inclusion of solutes in the superabsorbent polymer leading to an increased paramagnetism^16^. Precipitates may also form near superabsorbent polymers, leading to an increase in surface relaxation. The water molecules at the interface with the cementitious matrix can be the cause of a diffusive exchange with the water in the superabsorbent polymer particles, leading to a so-called intermediate exchange^26,27^. This causes the range of relaxation times found when studying superabsorbent polymer specimens. As the superabsorbent polymers’ signals are still pronounced during dry periods, entrained water is still available and this stored water can be released during dry periods, explaining the observed regain in mechanical properties found in literature^7,8^. The signal intensities of a reference sample stored at a relative humidity condition show the same results as if a dry sample is studied. When studying specimens containing superabsorbent polymers at relative humidity conditions, entrained water is found, especially at a high relative humidity of more than 90%. These graphs are found in Supporting Information.

In order to draw quantitative conclusions, the NMR signal needed to be calibrated to the mass scale readings of known water amounts. The increase in NMR signal intensity was linear compared to the amount of absorbed water in cementitious materials. The found calibration and trend line (Fig. S2) amounts to [NMR signal intensity [a.u] = 1.7·10^9^ · mass scale reading [g]] (R² = 0.986). With this calibration method, the amount of water seen by NMR could be calculated at any time during testing.

**Figure 2:**
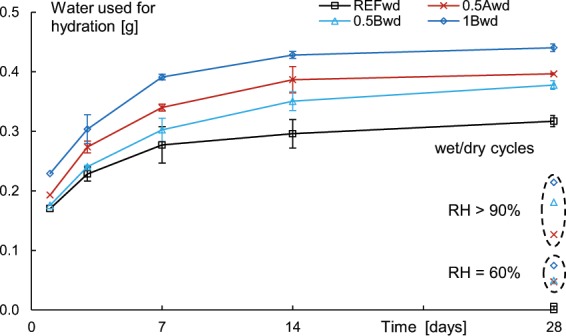
The calculated amount of water [g] used for hydration for all studied samples, stored in wet/dry cycles, at a relative humidity of more than 90%, and at a relative humidity of 60% as a function of healing time [days]. Specimens with superabsorbent polymers promote autogenous healing.

The NMR parameters in this study were used to only detect the non-chemically bound water. As part of the water is chemically bound during further hydration, the NMR signal intensity decreases. The difference between the measured NMR signal intensity and the mass scale readings thus points to the transition from non-chemically bound water to chemically bound water due to the further hydration, as seen in Fig. 2 for all studied mixtures and different healing conditions. The first measured values with NMR and mass scale readings always coincided and the subsequent values did not, pointing to the consummation of water for further hydration.

When studying the wet/dry cycles, i.e. the solid lines, the amount of water consumed for hydration increases with the number of healing cycles. The most healing occurred during the first 3 healing cycles, which is also reported in literature, as was observed by means of microscopic analysis^8,11^. All specimens containing superabsorbent polymer show a higher amount of water used for hydration. This is due to the availability of water during dry periods as well, promoting the formation of healing products. The specimens containing the same amount of SAP A and SAP B show the same trend with slightly more water used for SAP A samples. The best overall results are found in the 1B sample having 1 m% of SAP B. This will lead to the higher observed regain in mechanical properties^7,8^. The results show that the further hydration is promoted with 19% to 39% for 0.5B and 1B, respectively. This increase is noteworthy.

When studying the samples healed at relative humidity conditions, only the specimens with superabsorbent polymers showed an increase in mass compared to the initial testing prior to healing. Interestingly, the amount of water recorded with NMR and the mass scale readings were not the same for the samples with superabsorbent polymers after 28 days of healing. The moisture adsorbed by the superabsorbent polymers is thus used for further hydration. When calculating the amount of water used compared to the consumed water in a reference system healed in wet/dry cycling, the amount of promoted autogenous healing amounts to 15% to 24% in specimens containing superabsorbent polymers healed at 60% relative humidity, and 40% to 68% when the same specimens are healed at a relative humidity of more than 90%. This shows the beneficial feature of superabsorbent polymers to promote healing at relative humidity conditions. The healing is still half as if a specimen without superabsorbent polymers would be stored in wet/dry cycling conditions.

The results are supported by means of visual observance of the crack closure due to autogenous healing as studied by means of optical microscopy (Leica S8 APO mounted with a DFC 295 camera). The cracks in reference samples visually closed around 10% after 28 days exposure in wet/dry cycling. When being stored at high relative humidity, the visual closure was not noteworthy. After 28 days of healing, cracks in SAP specimens closed around 20% to 40% in wet/dry cycles, 5% to 10% at a relative humidity of more than 90%, and 1% to 5% at a relative humidity of 60%. These results are further supported by previous X-ray microtomography and neutron radiography results on analogous samples^9,10^.

To conclude, entrained water stored in superabsorbent polymers could be distinguished by means of NMR. A higher amount of water mass is absorbed in specimens containing superabsorbent polymers, due to the swelling in macro pores. During dry periods in wet/dry cycling, the specimens containing superabsorbent polymers show a larger amount of available free water to promote autogenous healing. The difference between the measured NMR signal intensity and the mass scale readings served as basis to determine the chemically bound water due to the further hydration. Most of the healing occurred in the first wet/dry cycles and is higher when superabsorbent polymers are added. The results show that the further hydration is promoted up to 39% when storing specimens in wet/dry cycling. No healing was observed in reference samples stored at relative humidity conditions. When adding superabsorbent polymers able to adsorb moisture from the environment, further hydration is noticed ranging up to 68% of the value of a reference sample healed in wet/dry cycling. This strain-hardening cementitious material with superabsorbent polymers is thus interesting to be applied as a self-healing material, either as basis material or as patch repair system. It is able to promote autogenous healing and thus interesting to be applied as construction material. These new research findings open up the path to model the material in terms of autogenous healing and to add information to the structural codes.

## Experimental Section

### Materials

Typical self-healing strain-hardening cementitious materials were studied (Table 1). These mixtures were based on previous obtained research and the mechanical properties can be found in literature^28^. Four different strain-hardening mixtures were investigated, being REF, 0.5 A, 0.5B and 1B. They all contain cement (CEM I 52.5 N from Holcim, Belgium), fly ash (Class F from OBBC, Belgium), silica sand (M34 with a D_50_ of 170 µm from Sibelco, Belgium), a polycarboxylate-type high-range water reducing agent (Glenium 51 with 35% conc. from BASF, Belgium), poly(vinyl alcohol) microfibers (oil-coated, 15 dtex, 8 mm length, 12 cN/dtex from Kuraray, Japan), water, and aside from the reference, a varying amount of superabsorbent polymer A or B (from BASF, Germany). SAP A is composed of sodium acrylate and acrylamide. This copolymer has a size of 100 ± 21 µm and is able to swell up to 305 ± 4 g/g in demineralized water, as determined with the filtration method^29^. SAP B with a size of 477 ± 53 µm and a swelling of 283 ± 2 g/g in demineralized water is a cross-linked potassium salt polyacrylate. At 60% relative humidity these superabsorbent polymers are able to adsorb 26% of their mass, and higher than 90% relative humidity 83% to 394% as determined with dynamic vapor sorption tests^30^. The SAP/cement ratio amounted to 0.5 m% and 1 m% relative to the cement weight. An additional amount of water was added to obtain the same workability. This corresponded to 30.5 g/g additional mixing water per SAP A and 8.9 g/g per SAP B. This entrained water is used for internal curing purposes and is not influencing the effective water-to-binder ratio.

**Table 1:** Studied strain-hardening mixtures with and without superabsorbent polymers [kg/m³].

Sample code	m% SAP [%]	SAP type	Cement [kg/m³]	Fly ash [kg/m³]	Sand [kg/m³]	Water [kg/m³]	Superplasticizer [kg/m³]	Fibers [kg/m³]	SAP [kg/m³]
REF	—	—	599	599	420	360	6.0	26	—
0.5 A	0.5	A	547	547	383	412	5.5	26	2.73
0.5B	0.5	B	582	582	408	375	5.8	26	2.91
1B	1	B	566	566	396	390	5.7	26	5.66

### Mixing procedure

The material was hand-mixed with a spatula. First, cement, fly ash and superabsorbent polymers were dry mixed for 60 s. The superplasticizer was dissolved in the total amount of mixing and entrained water and added to the mixture for 30 s at slow pace. The sand was added during continuous mixing for 30 s and rapid mixing for the next 30 s. After a resting period of 60 s the microfibers were added during 30 s followed by thorough mixing during 60 s.

### Storage conditions

The mixture was poured in cylindrical sample containers with a diameter of 27 mm and a height of 100 mm which were subsequently sealed. The samples were then stored at a temperature of 20 ± 2 °C until the age of 28 days.

### Sample preparation and cracking conditions

At 28 days, the samples were dry cut to remove the upper and lower part of the specimen. After sample cutting, the 40 mm high samples were then cracked in the middle by means of a Brazilian splitting test to a crack width of 200–250 µm, perpendicular to the cylindrical axis.

### Healing and testing conditions

Three different *ex-situ* healing conditions were studied. These were wet/dry cycling, a relative humidity of more than 90% (95 ± 5%) and a relative humidity of 60 ± 5%. The temperature was 20 ± 2 °C. One wet/dry cycle consisted of placing the sample for 1 hour in demineralized water and a subsequent 23 hours at a relative humidity of 60 ± 5%.

### NMR experimental parameters

A sample container containing a surface-dry specimen was positioned in the NMR setup and moved by means of a step motor. The external magnetic field was 0.8 T and corresponded to a frequency of 38 MHz. By placing the poles of the iron-cored electromagnet 50 mm apart from each other and adding a coil to create and receive radiofrequency (RF) fields, a field could be generated. A Faraday shield was added to the setup to allow quantitative NMR measurements. An Anderson gradient coil of 0.3 T/m was used, giving a resolution of 0.84 mm. Following parameters were used: 29.422 MHz center frequency, 30 µs pulse time, 180 µs echo time and 64 averages. During Carr-Purcell-Meiboom-Gill sequences, a 1000 ms repetition time and 128 echoes were used for the REF samples and 2000 ms and 2048 echoes for the superabsorbent polymer specimens. The recorded relaxation signal as a complex summation of decaying signals was transformed using Fast Laplace Inversion to obtain a distributed spectrum of relaxation times T_2_. The signals were then normalized to the highest recorded signal intensity for all specimens studied. Immediately before and after NMR measuring, the mass was determined by means of a mass scale with 0.001 g precision. The NMR measurements were performed prior and after the relative humidity healing of 28 days. During the wet/dry cycling, the NMR measurements were performed prior to the wet stage and immediately after the wet stage at 1, 3, 7, 14 and 28 cycles. The dry specimen was again scanned with NMR after the 28^th^ cycle to complete the dataset.

## Supplementary information

Supporting Information.

Supporting Information 2.

## Data Availability

All NMR signal intensity plots can be found as Supporting Information. The datasets generated during and/or analysed during the current study are available from the corresponding author on reasonable request.
