# Supplementary material for: Autogenous Healing in Cementitious Materials with Superabsorbent Polymers Quantified by Means of NMR
Source: Sci Rep. 2020 Jan 20;10:642. doi: 10.1038/s41598-020-57555-0 (PMC6971030; doi:10.1038/s41598-020-57555-0)
Supplement: Supplementary file 1 — Supporting Information. [file 41598_2020_57555_MOESM1_ESM.pdf]

Supporting Information

Title Quantified Autogenous Healing in Cementitious Materials with Superabsorbent Polymers

Didier Snoeck*, Leo Pel, Nele De Belie

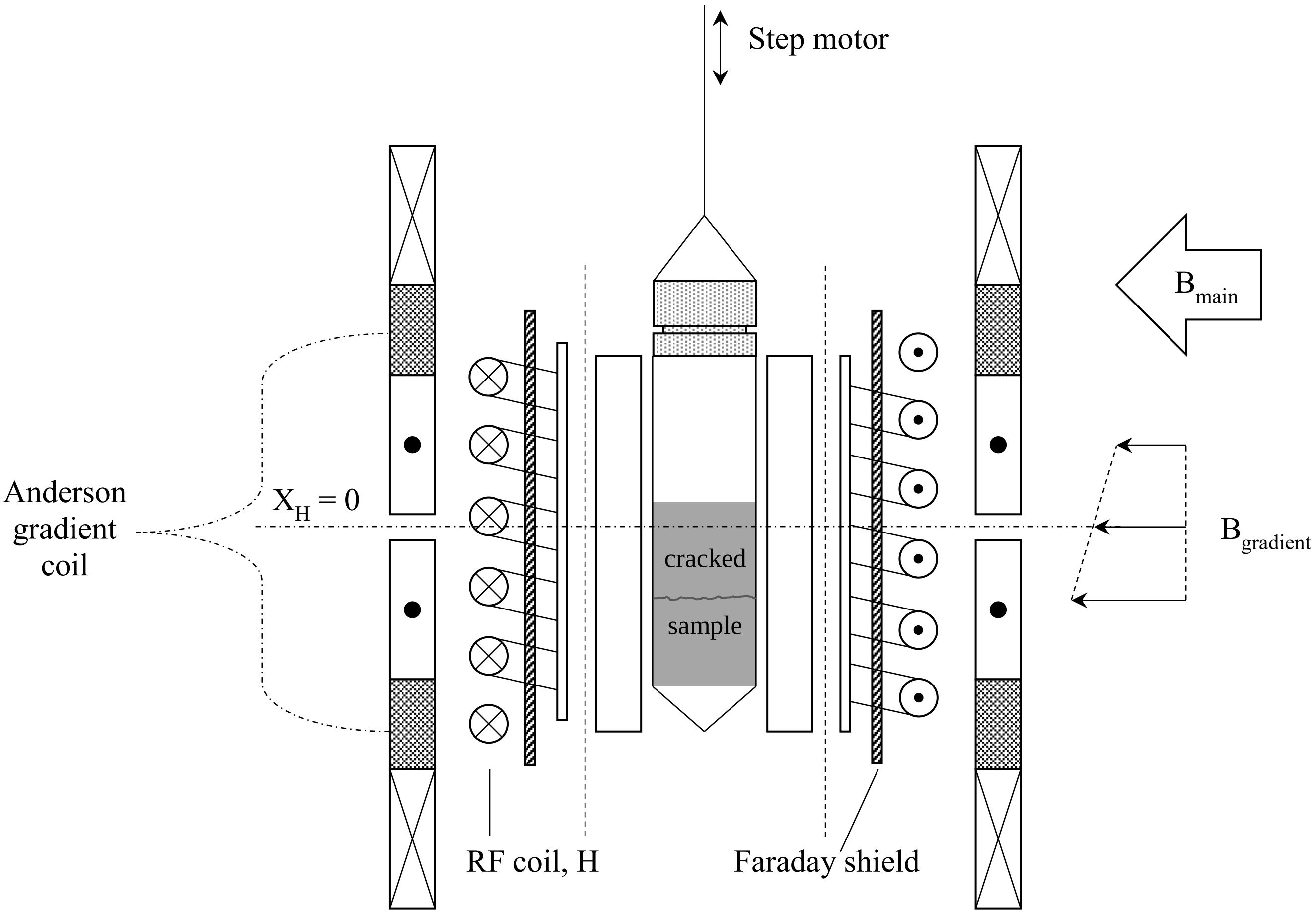

Figure S1. Used sample setup showing a sample put in container, which by means of a step motor is moved vertically through the NMR setup for measuring the moisture profiles during subsequent healing cycles.

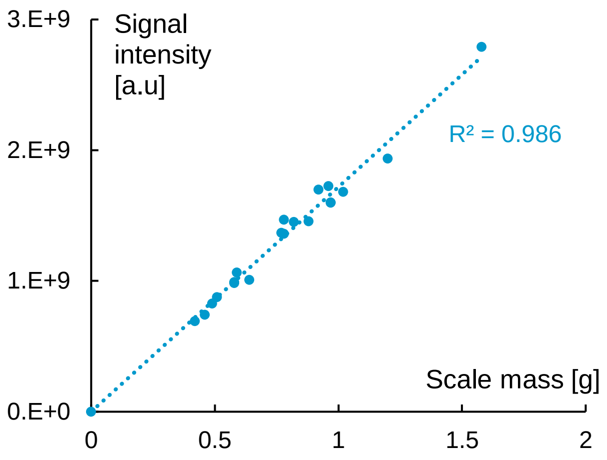

Figure S2. Validation of the water signal intensity obtained with NMR [a.u.] and the mass scale readings [g] showing a linear trend (R^2 = 0.986).

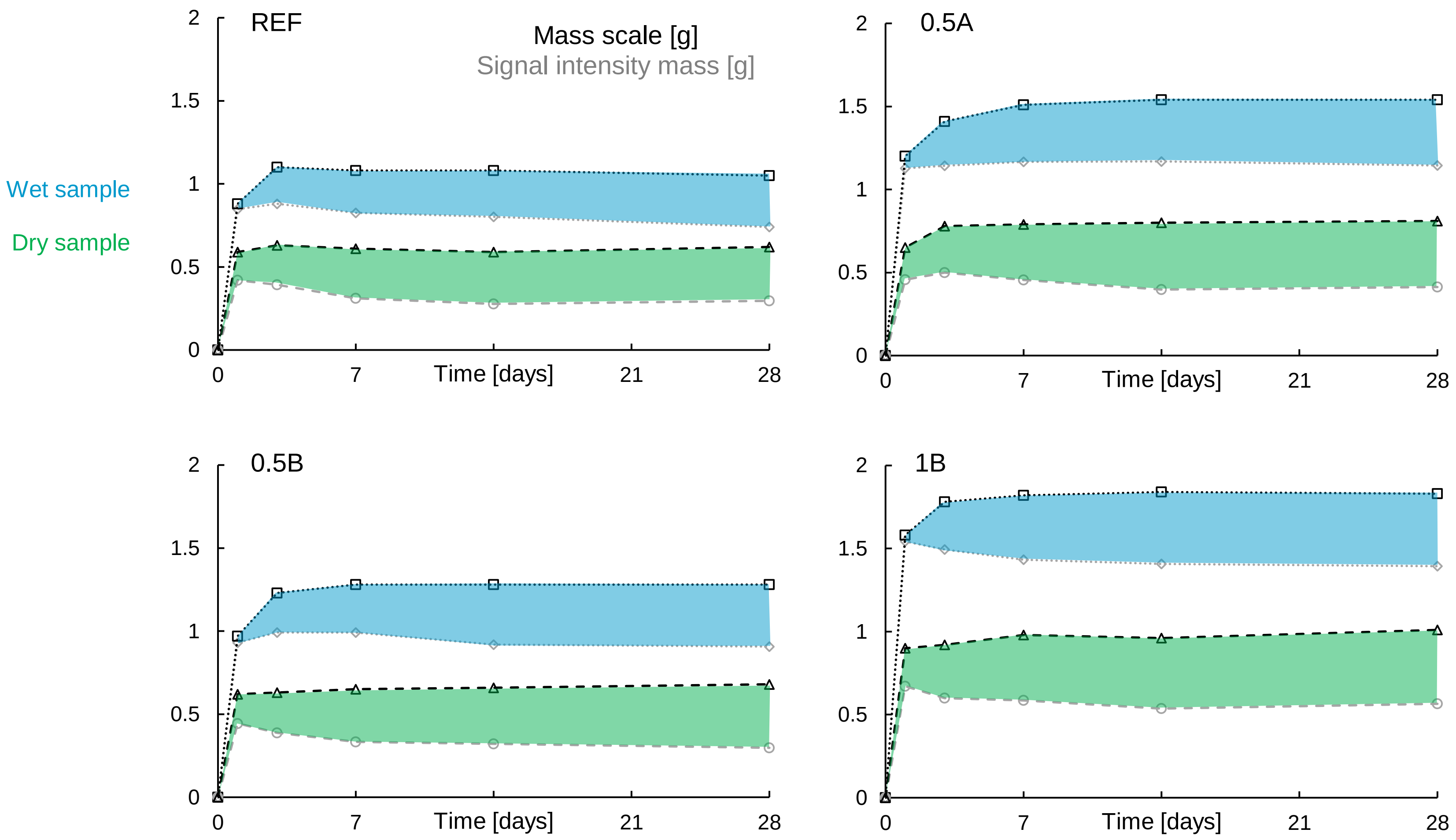

Figure S3. Mass scale versus signal intensity NMR readings for REF (top left), 0.5A (top right), 0.5B (bottom left) and 1B (bottom right) samples showing a difference in time for both wet and dry samples during wet/dry cycling.

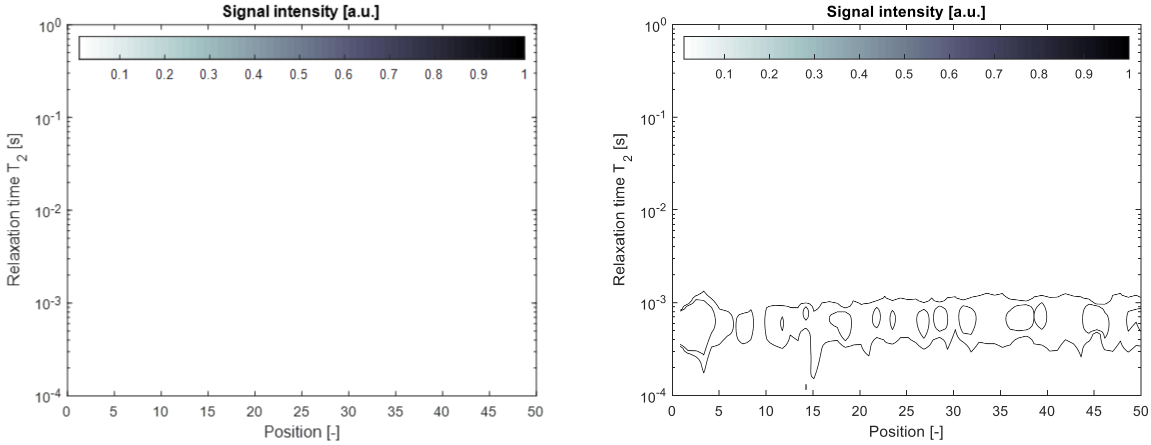

Figure S4. NMR signal intensity of an empty container (left) and with 0.01 a.u. boundary plot (right) showing no significant signal intensities and a different bandwidth compared to cementitious materials.

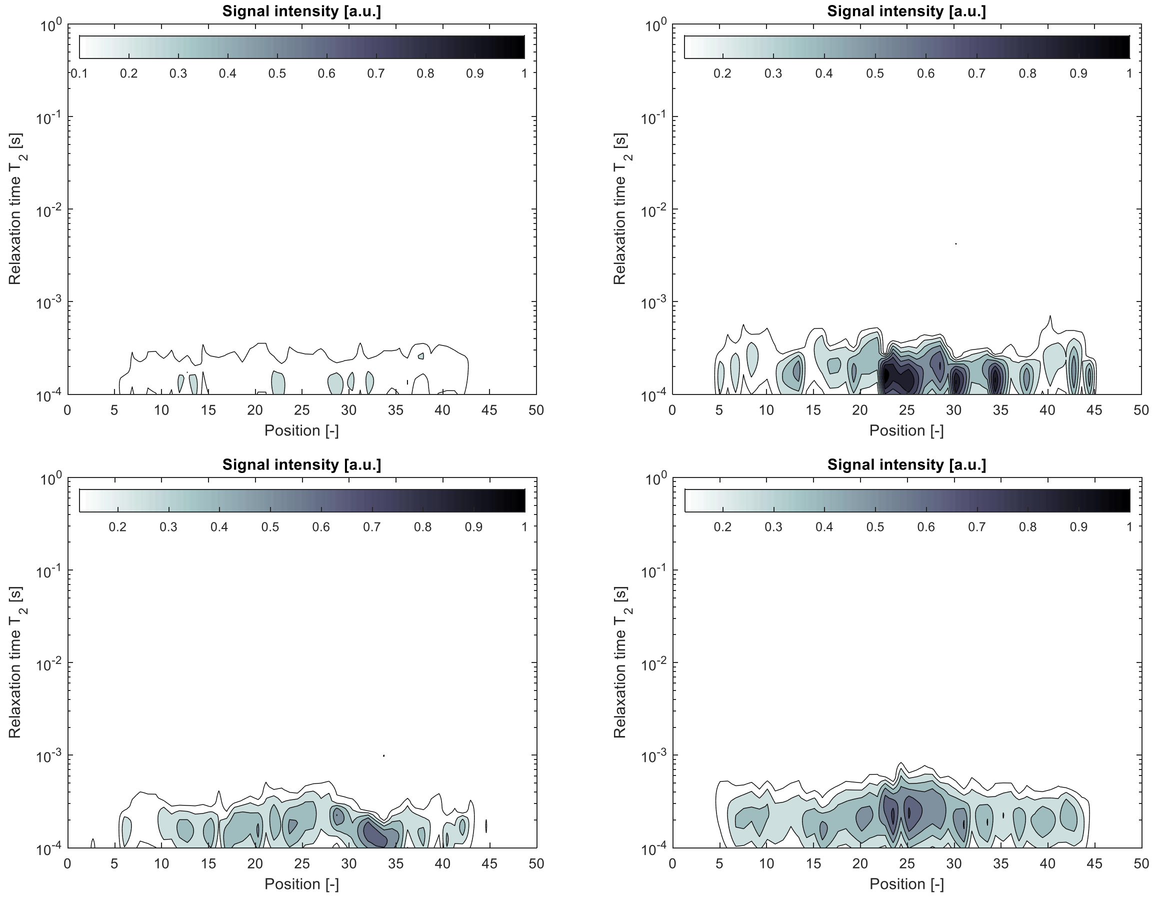

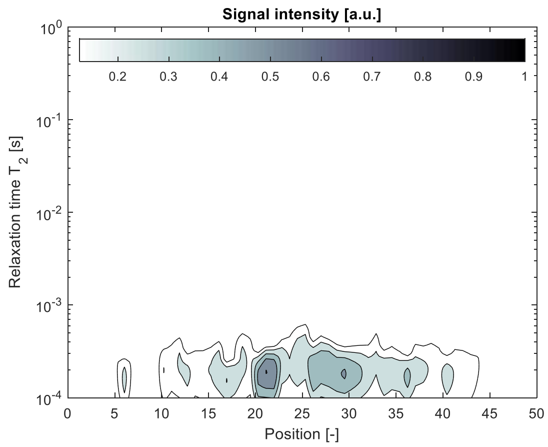
Signal intensity [a.u.]
0.2 0.3 0.4 0.5 0.6 0.7 0.8 0.9 1
Relaxation time T_2 [s]
10^0 10^{-1} 10^{-2} 10^{-3} 10^{-4}
0 5 10 15 20 25 30 35 40 45 50
Position [-]

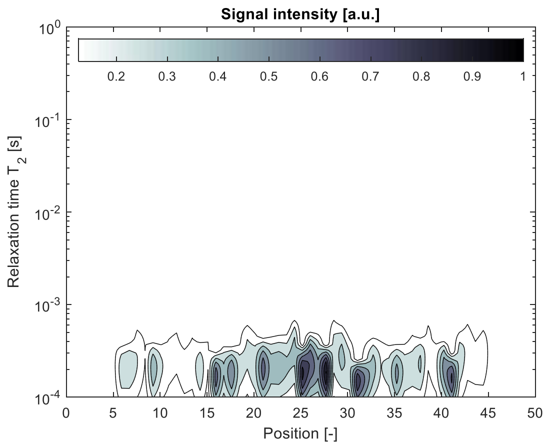
Signal intensity [a.u.]
0.2 0.3 0.4 0.5 0.6 0.7 0.8 0.9 1
Relaxation time T_2 [s]
10^0 10^{-1} 10^{-2} 10^{-3} 10^{-4}
0 5 10 15 20 25 30 35 40 45 50
Position [-]

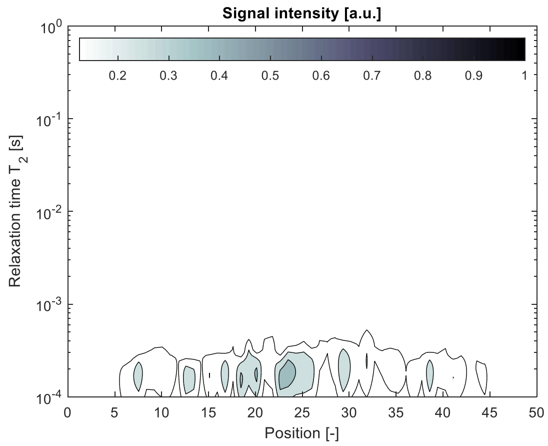
Signal intensity [a.u.]
0.2 0.3 0.4 0.5 0.6 0.7 0.8 0.9 1
Relaxation time T_2 [s]
10^0 10^{-1} 10^{-2} 10^{-3} 10^{-4}
0 5 10 15 20 25 30 35 40 45 50
Position [-]

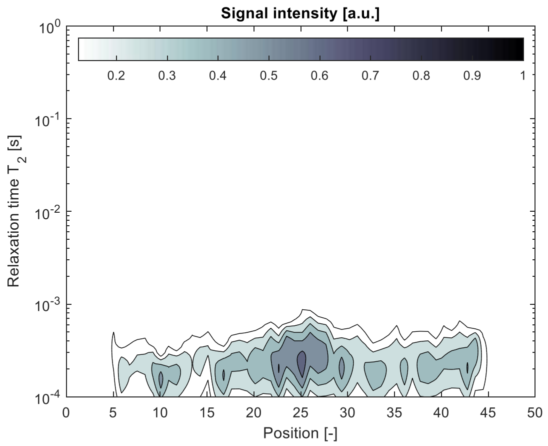
Signal intensity [a.u.]
0.2 0.3 0.4 0.5 0.6 0.7 0.8 0.9 1
Relaxation time T_2 [s]
10^0 10^{-1} 10^{-2} 10^{-3} 10^{-4}
0 5 10 15 20 25 30 35 40 45 50
Position [-]

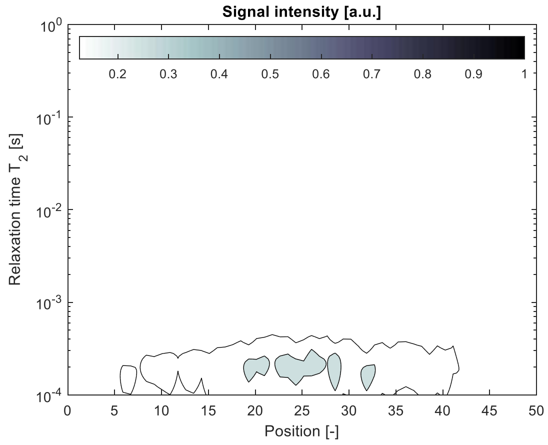
Signal intensity [a.u.]
0.2 0.3 0.4 0.5 0.6 0.7 0.8 0.9 1
Relaxation time T_2 [s]
10^0 10^{-1} 10^{-2} 10^{-3} 10^{-4}
0 5 10 15 20 25 30 35 40 45 50
Position [-]

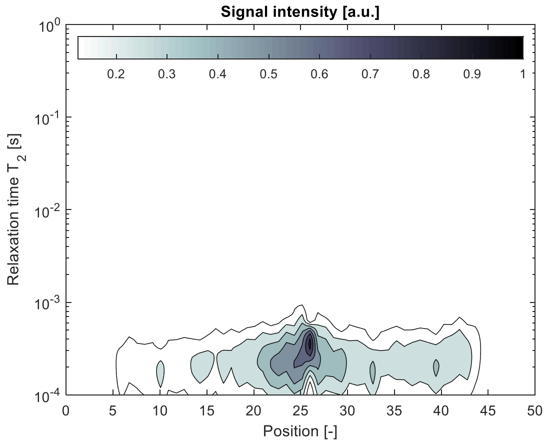
Signal intensity [a.u.]
0.2 0.3 0.4 0.5 0.6 0.7 0.8 0.9 1
Relaxation time T_2 [s]
10^0 10^{-1} 10^{-2} 10^{-3} 10^{-4}
0 5 10 15 20 25 30 35 40 45 50
Position [-]

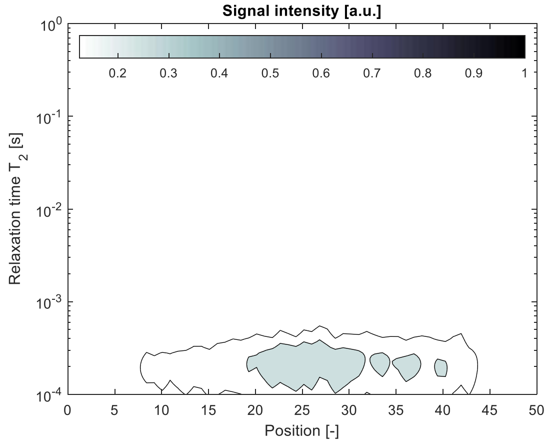
Signal intensity [a.u.]
0.2 0.3 0.4 0.5 0.6 0.7 0.8 0.9 1
Relaxation time T_2 [s]
10^0 10^{-1} 10^{-2} 10^{-3} 10^{-4}
0 5 10 15 20 25 30 35 40 45 50
Position [-]

Figure S5. NMR signal intensity of a reference sample subjected to wet dry cycles giving the dry (left-hand side) and wet (right-hand side) states after 1, 3, 7, 14 and 28 wet/dry cycles. The last left picture shows the dry state after the 28th cycle.

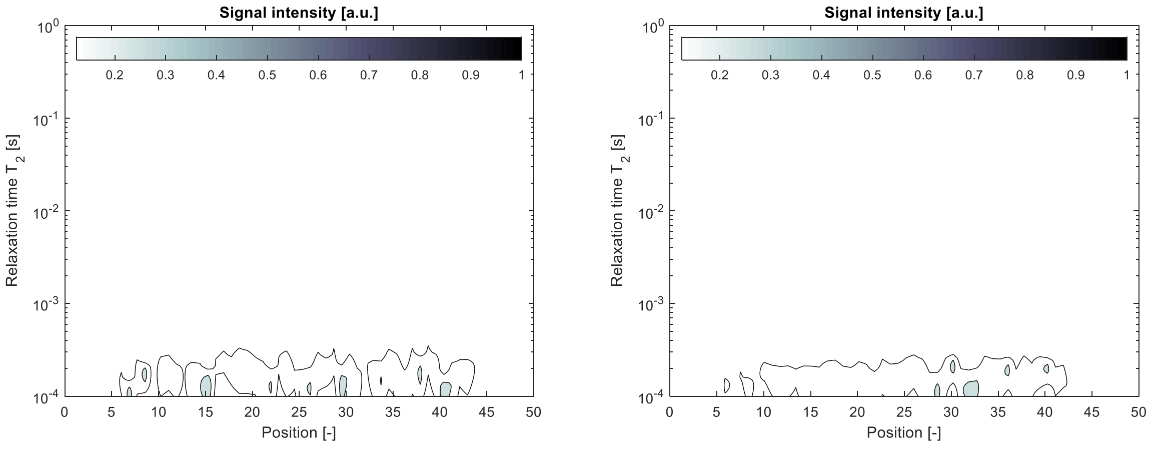

Figure S6. NMR signal intensity of a reference sample before (left) and after (right) being subjected to a relative humidity of more than 90% for 28 days.

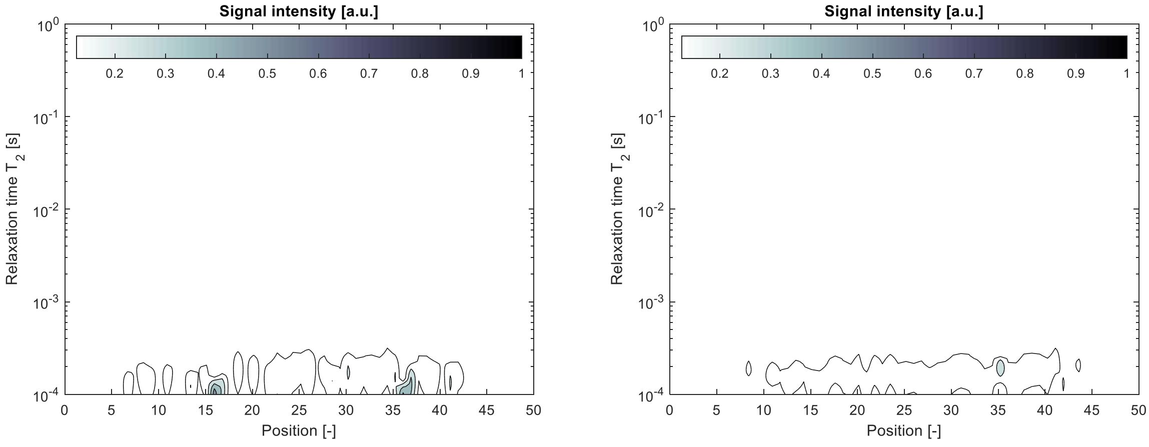

Figure S7. NMR signal intensity of a reference sample before (left) and after (right) being subjected to a relative humidity of 60% for 28 days.

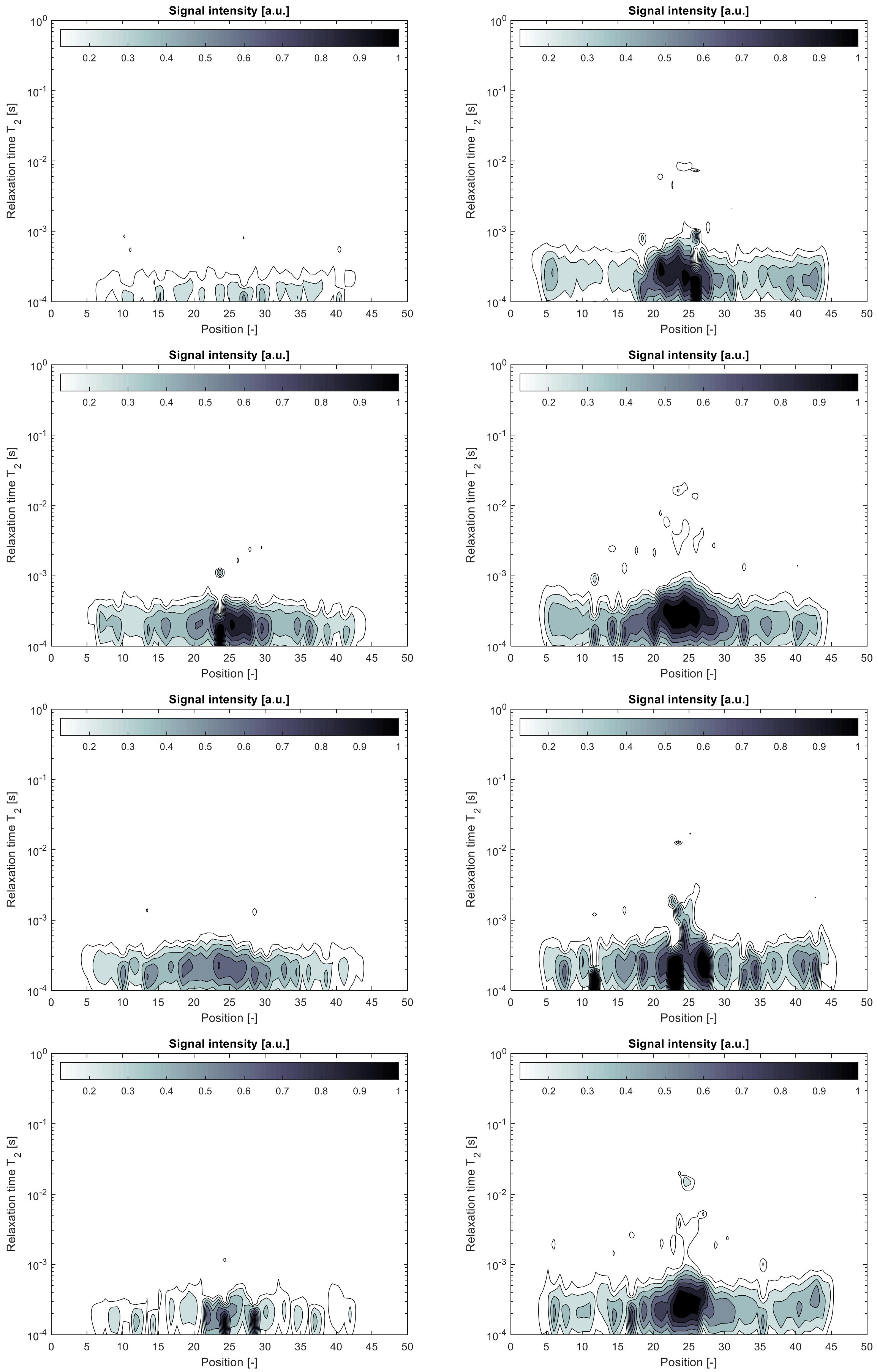

Signal intensity [a.u.]
0.2 0.3 0.4 0.5 0.6 0.7 0.8 0.9 1
Relaxation time T_2 [s]
10^0 10^{-1} 10^{-2} 10^{-3} 10^{-4}
Position [-]
0 5 10 15 20 25 30 35 40 45 50

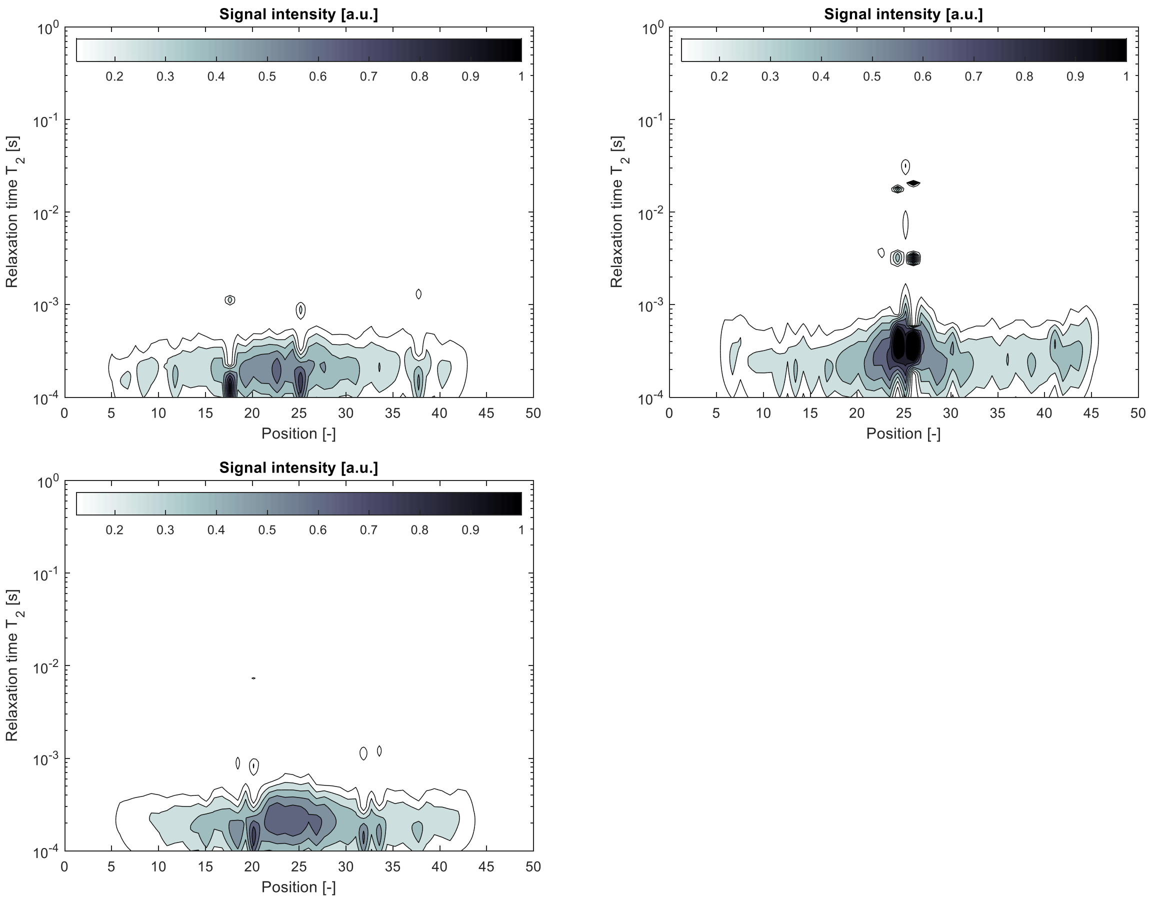

Figure S8. NMR signal intensity of the 0.5A sample subjected to wet dry cycles giving the dry (left-hand side) and wet (right-hand side) states after 1, 3, 7, 14 and 28 wet/dry cycles. The last left picture shows the dry state after the 28th cycle.

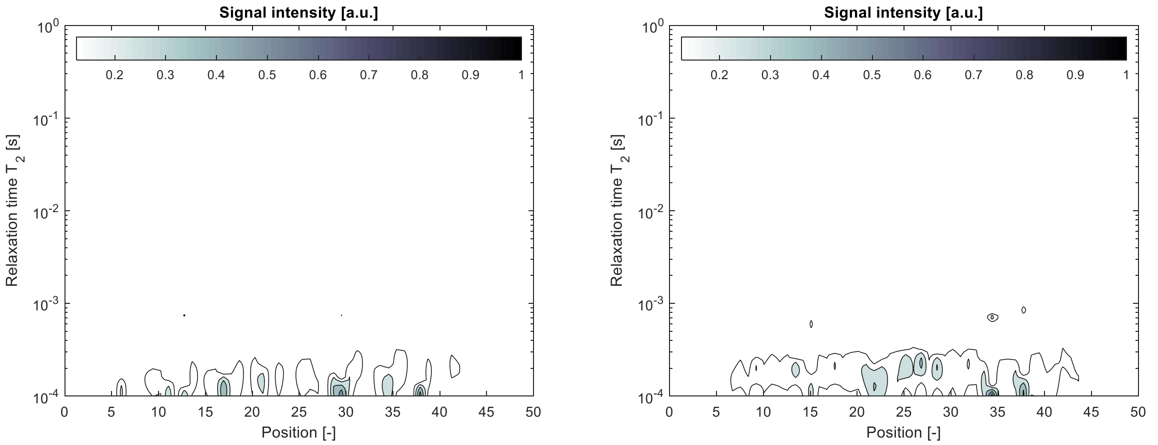

Figure S9. NMR signal intensity of the 0.5A sample before (left) and after (right) being subjected to a relative humidity of more than 90% for 28 days.

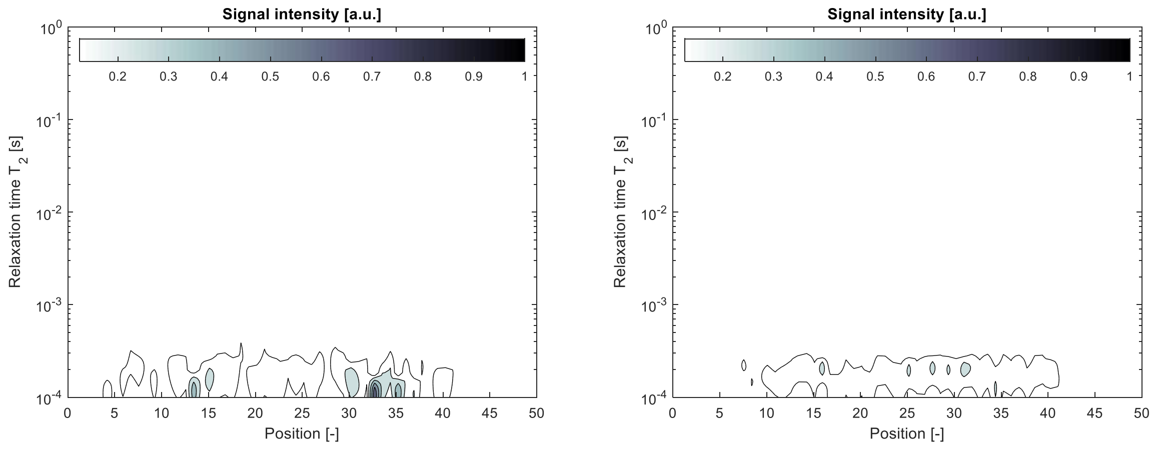

Figure S10. NMR signal intensity of the 0.5A sample before (left) and after (right) being subjected to a relative humidity of 60% for 28 days.

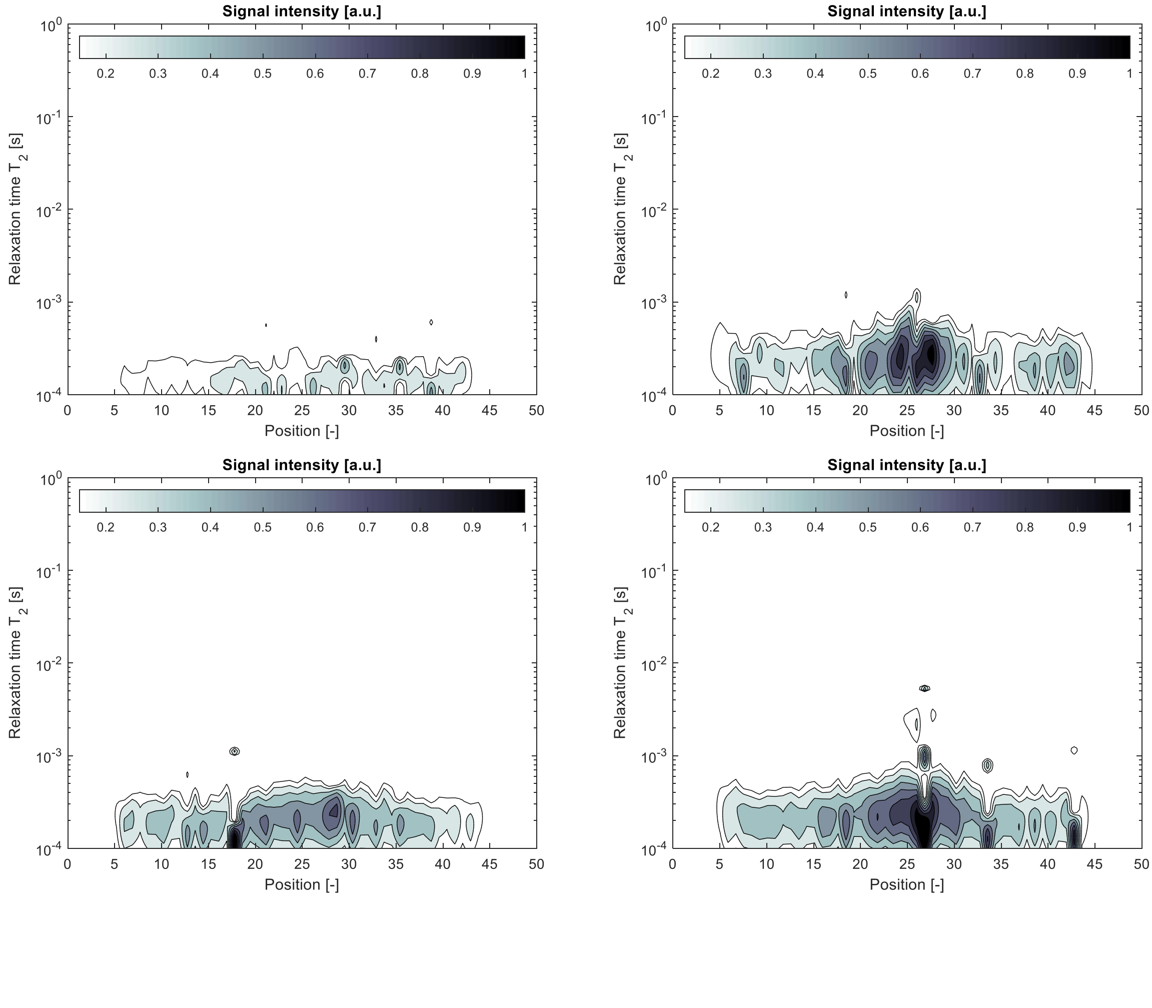

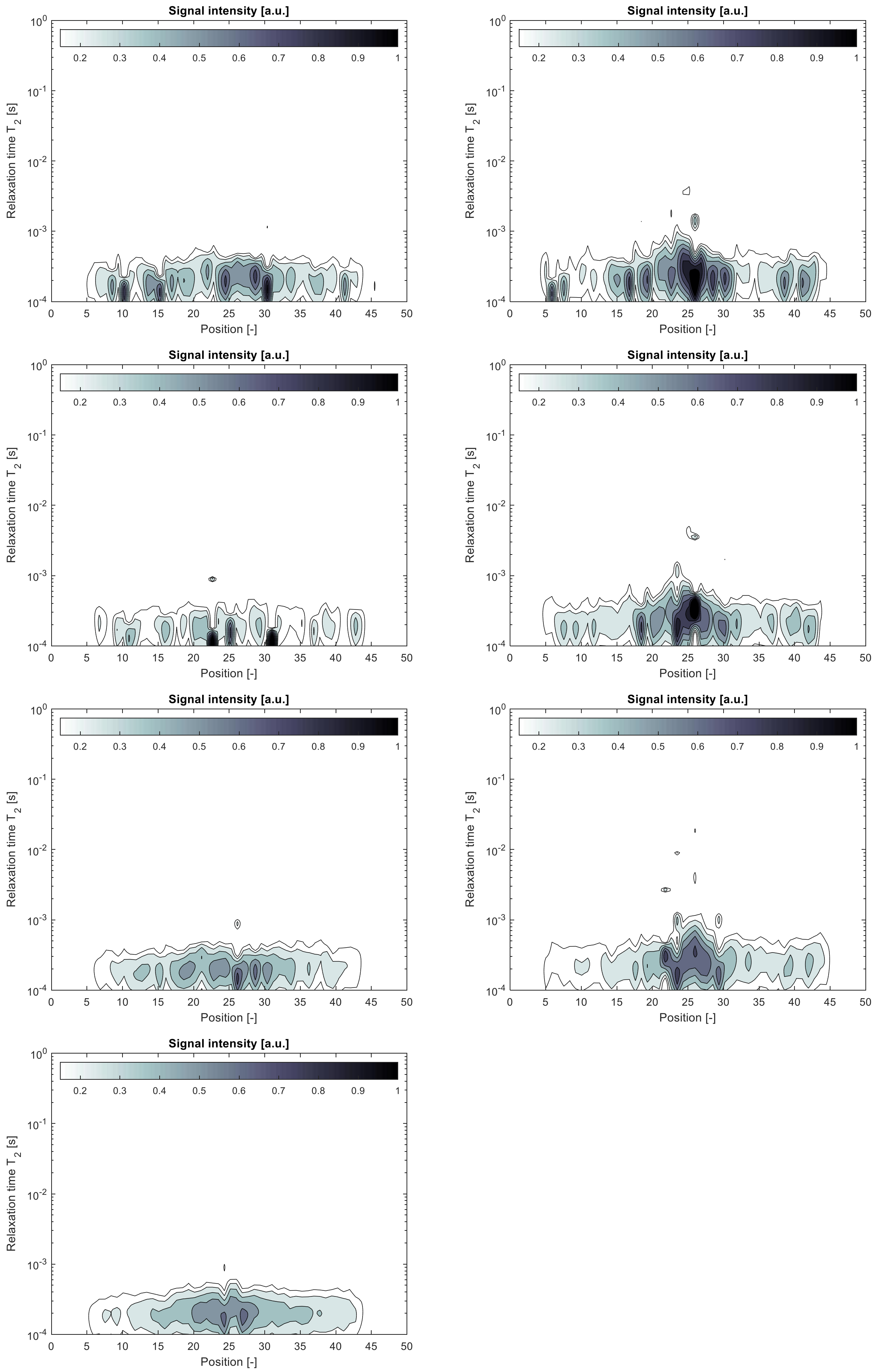
Signal intensity [a.u.]
0.2 0.3 0.4 0.5 0.6 0.7 0.8 0.9 1
Relaxation time T_2 [s]
10^0 10^{-1} 10^{-2} 10^{-3} 10^{-4}
0 5 10 15 20 25 30 35 40 45 50
Position [-]

Figure S11. NMR signal intensity of the 0.5B sample subjected to wet dry cycles giving the dry (left-hand side) and wet (right-hand side) states after 1, 3, 7, 14 and 28 wet/dry cycles. The last left picture shows the dry state after the 28th cycle.

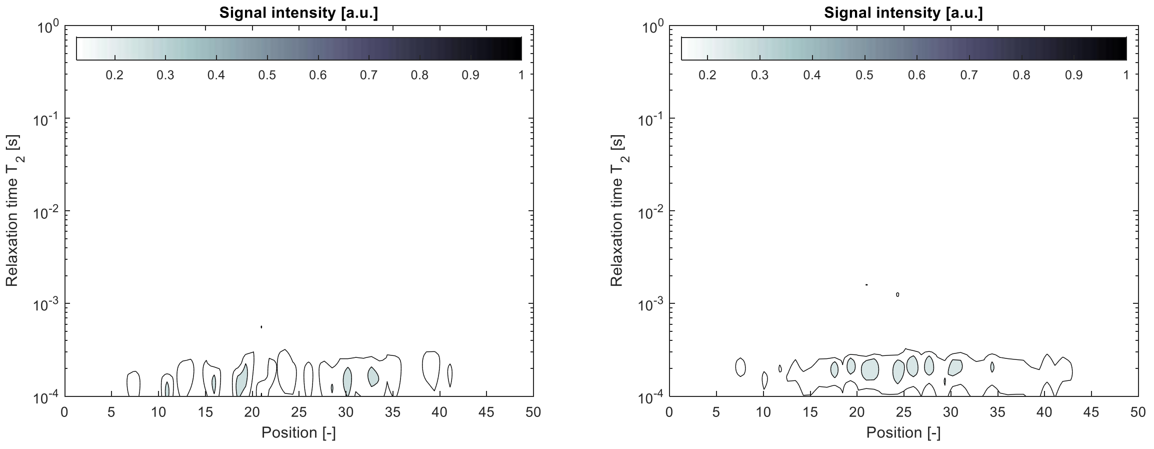

Figure S12. NMR signal intensity of the 0.5B sample before (left) and after (right) being subjected to a relative humidity of more than 90% for 28 days.

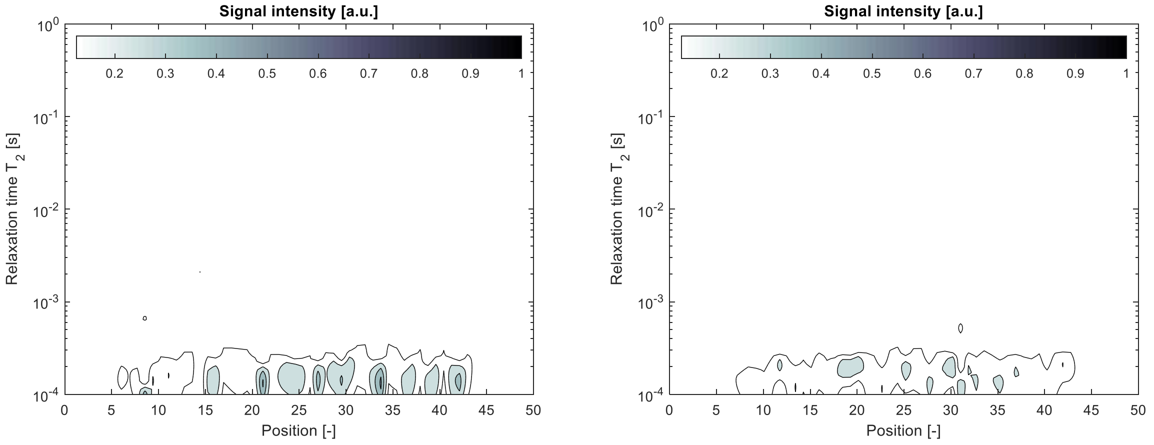

Figure S13. NMR signal intensity of the 0.5B sample before (left) and after (right) being subjected to a relative humidity of 60% for 28 days.

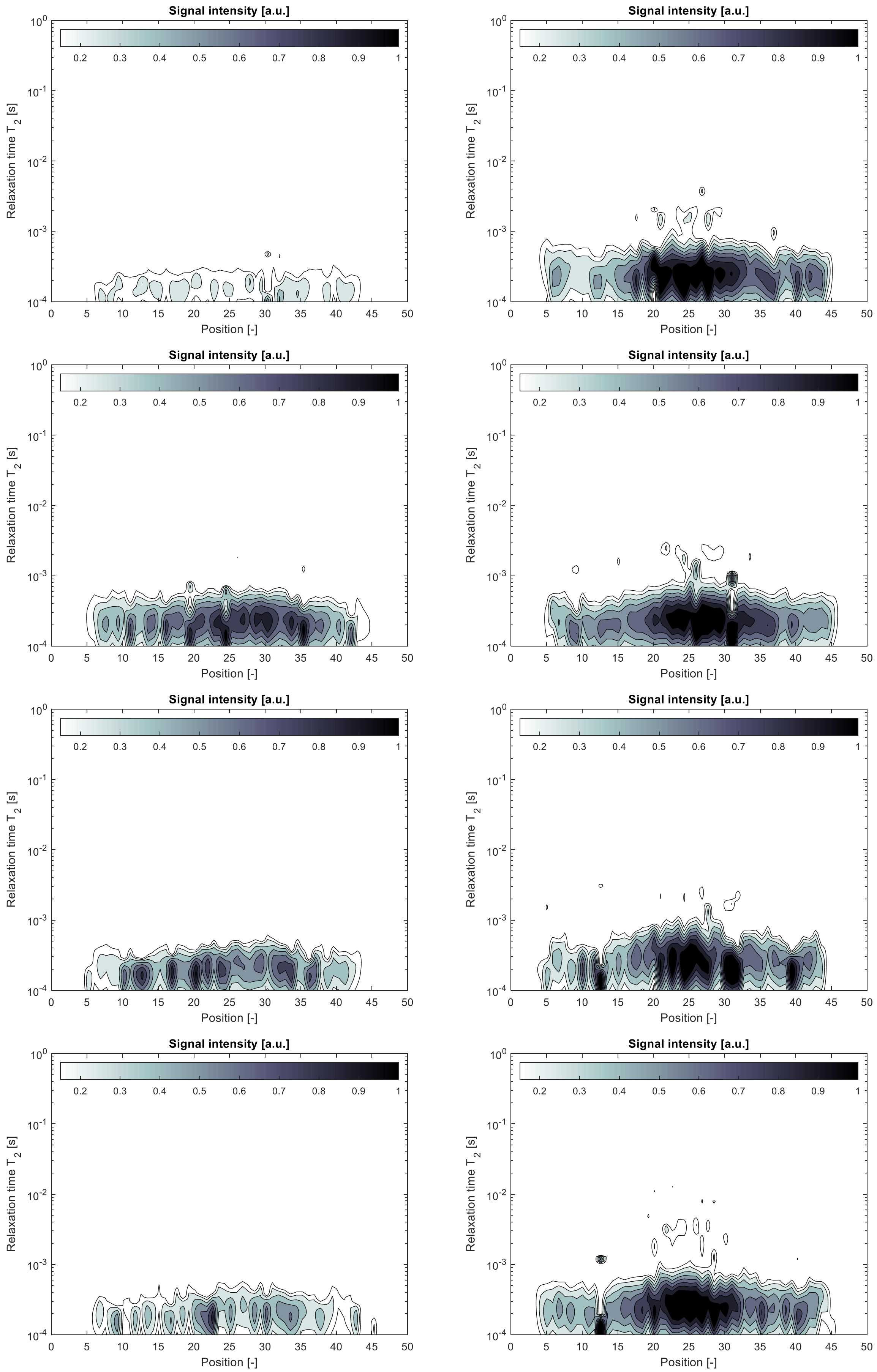
Signal intensity [a.u.]
0.2 0.3 0.4 0.5 0.6 0.7 0.8 0.9 1
Relaxation time T_2 [s]
10^0 10^{-1} 10^{-2} 10^{-3} 10^{-4}
Position [-]
0 5 10 15 20 25 30 35 40 45 50

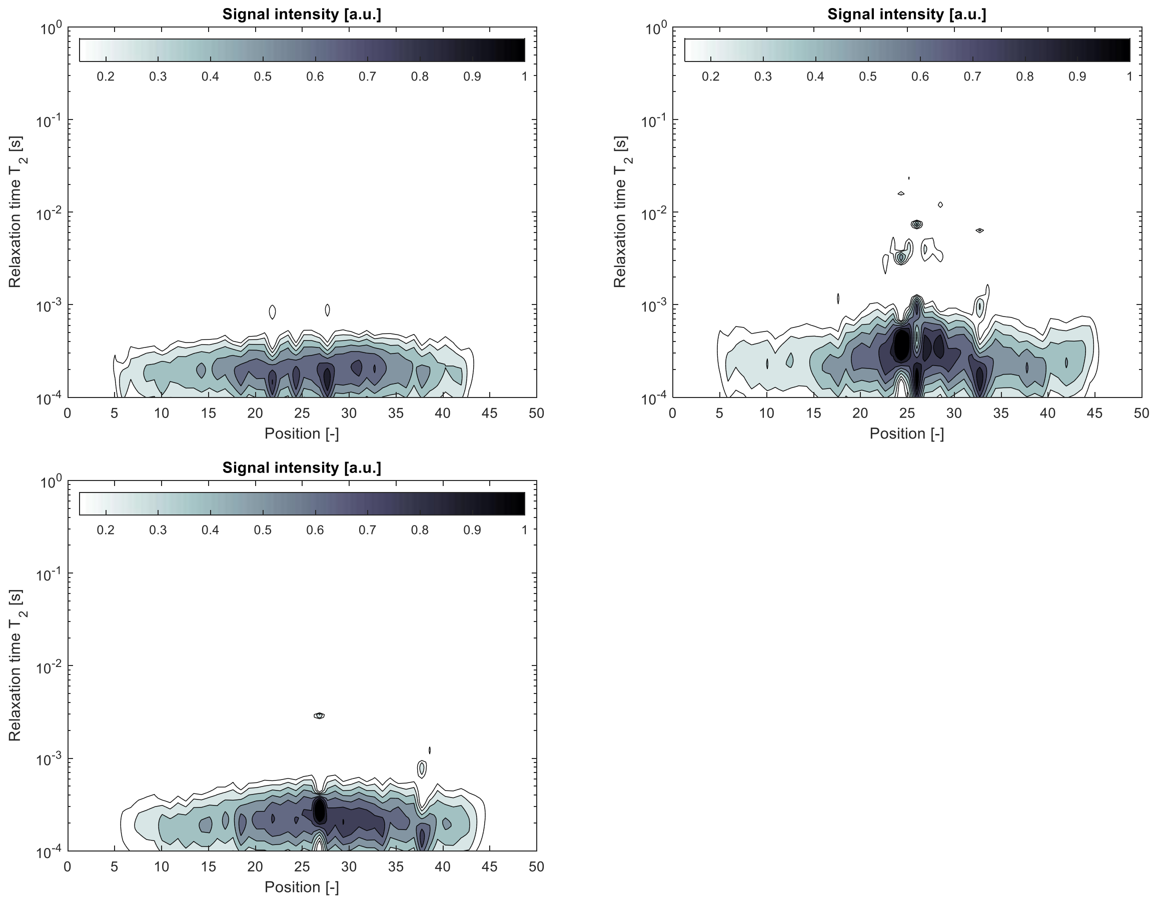

Figure S14. NMR signal intensity of the 1B sample subjected to wet dry cycles giving the dry (left-hand side) and wet (right-hand side) states after 1, 3, 7, 14 and 28 wet/dry cycles. The last left picture shows the dry state after the 28th cycle.

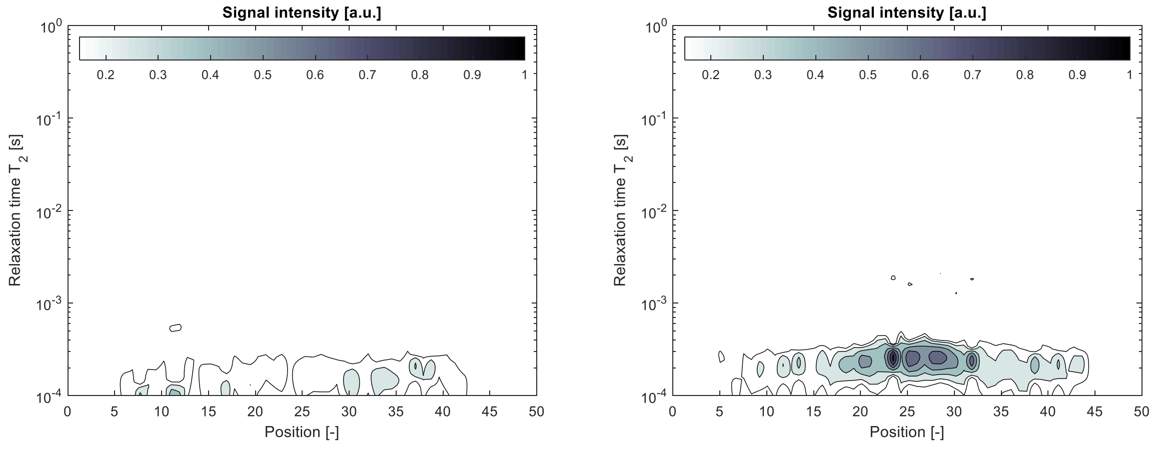

Figure S15. NMR signal intensity of the 1B sample before (left) and after (right) being subjected to a relative humidity of more than 90% for 28 days.

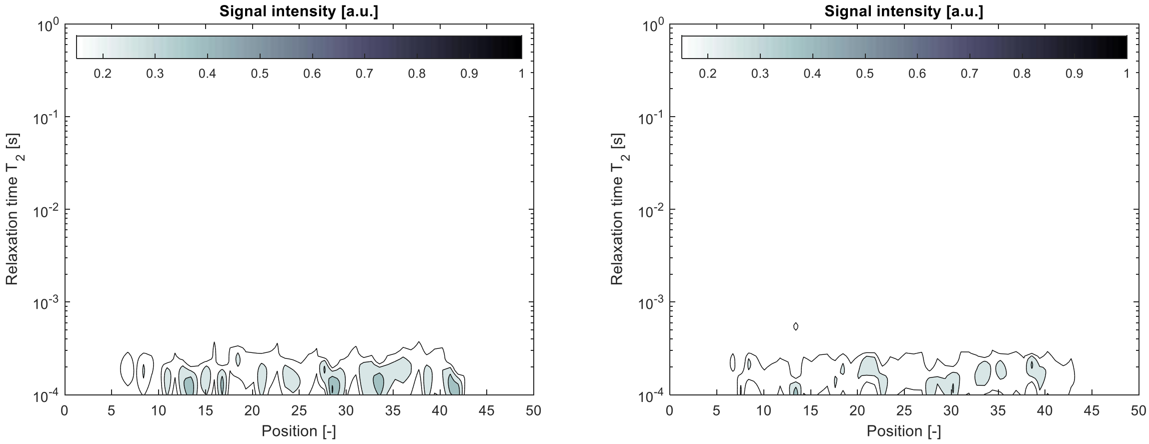

Figure S16. NMR signal intensity of the 1B sample before (left) and after (right) being subjected to a relative humidity of 60% for 28 days.
