# Supplementary material for: Autogenous Healing in Cementitious Materials with Superabsorbent Polymers Quantified by Means of NMR
Source: Sci Rep. 2020 Jan 20;10:642. doi: 10.1038/s41598-020-57555-0 (PMC6971030; doi:10.1038/s41598-020-57555-0)
Supplement: Supplementary file 2 — Supporting Information 2. [file 41598_2020_57555_MOESM2_ESM.docx]

Supporting Information

**Title** Quantified Autogenous Healing in Cementitious Materials with Superabsorbent Polymers

Didier Snoeck*, Leo Pel, Nele De Belie

Faraday shield

RF coil, H

X_H_ = 0

Anderson gradient coil

B_gradient_

B_main_

cracked

sample

Step motor

**Figure S1.** Used sample setup showing a sample put in container, which by means of a step motor is moved vertically through the NMR setup for measuring the moisture profiles during subsequent healing cycles.

**Figure S2.** Validation of the water signal intensity obtained with NMR [a.u.] and the mass scale readings [g] showing a linear trend (R² = 0.986).


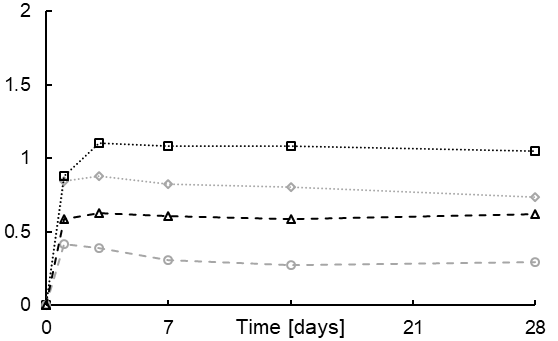

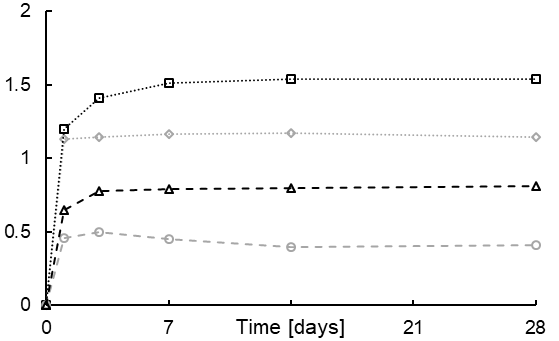

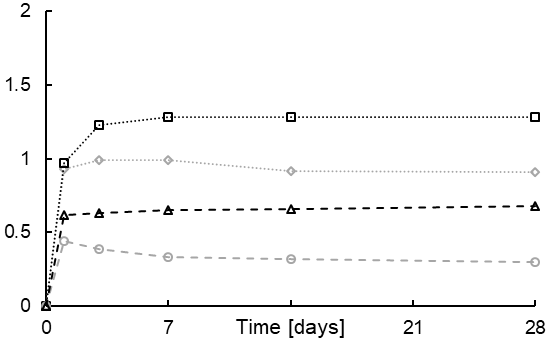

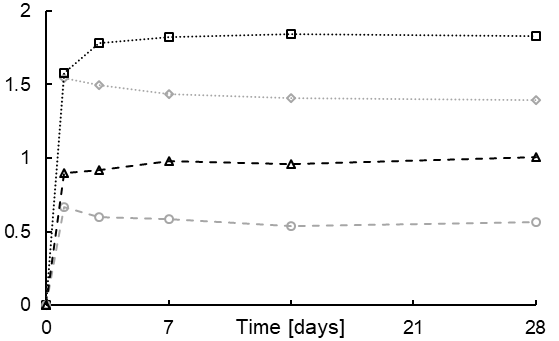


REF 0.5A

0.5B 1B

Mass scale [g]

Signal intensity mass [g]

Wet sample

Dry sample

**Figure S3.** Mass scale versus signal intensity NMR readings for REF (top left), 0.5A (top right), 0.5B (bottom left) and 1B (bottom right) samples showing a difference in time for both wet and dry samples during wet/dry cycling.


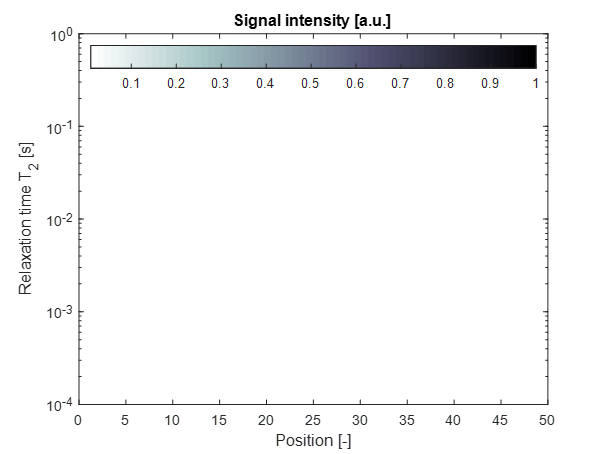


**Figure S4.** NMR signal intensity of an empty container (left) and with 0.01 a.u. boundary plot (right) showing no significant signal intensities and a different bandwidth compared to cementitious materials.

**Figure S5.** NMR signal intensity of a reference sample subjected to wet dry cycles giving the dry (left-hand side) and wet (right-hand side) states after 1, 3, 7, 14 and 28 wet/dry cycles. The last left picture shows the dry state after the 28^th^ cycle.

**Figure S6.** NMR signal intensity of a reference sample before (left) and after (right) being subjected to a relative humidity of more than 90% for 28 days.

**Figure S7.** NMR signal intensity of a reference sample before (left) and after (right) being subjected to a relative humidity of 60% for 28 days.

**Figure S8.** NMR signal intensity of the 0.5A sample subjected to wet dry cycles giving the dry (left-hand side) and wet (right-hand side) states after 1, 3, 7, 14 and 28 wet/dry cycles. The last left picture shows the dry state after the 28^th^ cycle.

**Figure S9.** NMR signal intensity of the 0.5A sample before (left) and after (right) being subjected to a relative humidity of more than 90% for 28 days.

**Figure S10.** NMR signal intensity of the 0.5A sample before (left) and after (right) being subjected to a relative humidity of 60% for 28 days.

**Figure S11.** NMR signal intensity of the 0.5B sample subjected to wet dry cycles giving the dry (left-hand side) and wet (right-hand side) states after 1, 3, 7, 14 and 28 wet/dry cycles. The last left picture shows the dry state after the 28^th^ cycle.

**Figure S12.** NMR signal intensity of the 0.5B sample before (left) and after (right) being subjected to a relative humidity of more than 90% for 28 days.

**Figure S13.** NMR signal intensity of the 0.5B sample before (left) and after (right) being subjected to a relative humidity of 60% for 28 days.

**Figure S14.** NMR signal intensity of the 1B sample subjected to wet dry cycles giving the dry (left-hand side) and wet (right-hand side) states after 1, 3, 7, 14 and 28 wet/dry cycles. The last left picture shows the dry state after the 28^th^ cycle.

**Figure S15.** NMR signal intensity of the 1B sample before (left) and after (right) being subjected to a relative humidity of more than 90% for 28 days.

**Figure S16.** NMR signal intensity of the 1B sample before (left) and after (right) being subjected to a relative humidity of 60% for 28 days.
